# Bouldering psychotherapy is more effective in the treatment of depression than physical exercise alone: results of a multicentre randomised controlled intervention study

**DOI:** 10.1186/s12888-020-02518-y

**Published:** 2020-03-12

**Authors:** Nina Karg, Lisa Dorscht, Johannes Kornhuber, Katharina Luttenberger

**Affiliations:** 1 Centre for Health Services Research in Medicine, Department of Psychiatry and Psychotherapy, Friedrich-Alexander-Universität Erlangen-Nürnberg (FAU), Schwabachanlage 6, 91054 Erlangen, Germany; 2 Department of Psychiatry and Psychotherapy, Friedrich-Alexander-Universität Erlangen-Nürnberg (FAU), Schwabachanlage 6, 91054 Erlangen, Germany

**Keywords:** Depression, Physical exercise, Bouldering, Psychotherapy

## Abstract

**Background:**

Recent scientific studies have suggested that climbing/bouldering is effective in alleviating depression when the comparison group was a waitlist control group, even when physical activity and other therapeutic approaches were controlled for. In the present study, we aimed to investigate the effectiveness of a manualised psychotherapeutic bouldering intervention for depressed individuals, compared with an active control group performing physical exercise alone.

**Methods:**

In a multicentre randomised controlled intervention trial, 133 outpatients with depression were assigned to either a bouldering psychotherapy (BPT) group or a home-based supervised exercise programme (EP). Severity of depression as the primary outcome was assessed at baseline and directly after a ten-week intervention period using the Montgomery–Åsberg Depression Rating Scale (MADRS). Secondary outcomes included anxiety, coping skills, self-esteem, body image, and interpersonal sensitivity. We applied t-tests to test for differences within the groups (t0 vs. t1) and between the BPT and the EP and a multiple regression analysis with the post-intervention MADRS score as the dependent variable. The robustness of estimates was investigated with a sensitivity analyses.

**Results:**

Patients in the BPT group showed a significantly larger decrease in depression scores compared with the EP on the MADRS (drop of 8.4 vs. 3.0 points, *p* = .002, Cohen’s *d* = 0.55). In the confounder-adjusted regression analyses, group allocation was found to be the only significant predictor of the post-intervention MADRS score (*β* = − 5.60, *p* = .001) besides the baseline MADRS score. Further significant differences in change scores between the BPT and the EP were found for anxiety (*p* = .046, *d* = 0.35), body image (*p* = .018, *d* = 0.42), and global self-esteem (*p* = .011, *d* = 0.45).

**Conclusions:**

The study provides evidence that the manualised BPT is not only effective in alleviating depressive symptoms but even goes beyond the effect of mere physical exercise. Based on these findings, the BPT should be considered as a complementary therapeutic approach.

**Trial registration:**

Trial identification number: ISRCTN12457760: Study KuS (Klettern und Stimmung - Climbing and Mood) combined boulder and psychotherapy against depression, registered retrospectively on July 26th, 2017.

## Background

Worldwide, only one out of six individuals suffering from depression receives minimally adequate treatment, a finding that reveals a significant therapeutic supply gap in the mental health care system [1]. Besides a lack of therapists and thus long waiting times for a therapy place, a missing belief in the effectiveness of classical psychotherapeutic interventions as well as stigma play a major role when it comes to actively seek help for the treatment of mental problems [2]. Hence, there is a need for novel and complementary treatment options that exceed traditional therapeutic interventions. As such an alternative approach, physical activity has been found to be effective for the treatment of depression. Previous research has found antidepressant effects for various modes of exercise, ranging from endurance and aerobic exercise such as walking, running, and cycling [3–6] to resistance and strength training [7, 8]. There is further scientific evidence of an association between low-intensity mindfulness-based techniques such as Yoga [9–11], Thai Chi, and Qigong [12–14], and an improvement in depressive symptoms. With moderate to large effect sizes (Cohen’s *d* between 0.62 and 0.82), the antidepressant effect of physical exercise has turned out to be comparable to psychotherapy and antidepressant psycho-pharmaceuticals [6, 8, 15, 16]. Consequently, both the NICE guidelines [17] and the German guidelines for the treatment of depression [18] have included the recommendation to implement physical activity as a complementary therapeutic method in the standard treatment of depression. Though the efficacy of physical activity is widely accepted, it remains largely unclear which mode and intensity of exercise is most promising in alleviating depressive symptoms. While some studies have demonstrated that resistance exercise is superior to aerobic exercise [15], others have indicated stronger long-term effects of aerobic endurance training compared with strength training [19]. Regarding the intensity of training, the effectiveness of low-intensity yoga-based stretching exercises was found to be comparable to vigorous intensity aerobic training in treating mild to moderate depression [20]. A special mode of exercise that has gained increased attention in clinical practice lately and has already been applied as part of the overall treatment plan in several clinics involves bouldering, which is defined as climbing to moderate heights without the use of ropes or harnesses [21, 22]. Recent research has shown positive effects of climbing/bouldering not only on various health problems [23–28] but also on mental disorders such as anxiety disorders [29], ADHD [29, 30], and eating disorders [29]. Several studies have indicated improvements related to climbing/bouldering in a number of domains that are believed to play an important role in the emergence and maintenance of depression, such as cognitive abilities [21, 29], self-confidence, self-esteem, self-efficacy [29, 31], and social skills [21, 29]. In line with these findings, there were some first indications that climbing and bouldering are effective in reducing depressive symptoms [21, 29, 32, 33]. However, conclusions regarding bouldering as an effective treatment for depression must be drawn with caution because existing studies are often limited by methodological problems such as small sample sizes, the use of unstandardised psychometric measures, no randomisation, or even the absence of any control groups [34]. Our work group [35, 36] conducted a randomised waitlist-controlled pilot study to investigate the effectiveness of an eight-week bouldering psychotherapeutic intervention on depressive symptoms in individuals with depression. Participants in the intervention group showed a significantly greater reduction in depressive symptoms (from a moderate to a mild severity level, Cohen’s *d* = 0.77) as well as improvements in a number of other mental health outcomes (i.e. anxiety and self-management) compared with the waitlist control group. We controlled for the confounding effect of general physical activity with the use of accelerometres. However, no direct comparison between the bouldering therapy and a proper sports/physical exercise programme (i.e. in the form of a fitness workout) was drawn. To follow up on this idea, the main aim of the current study called StudyKuS (Studie**KuS** – ‘**K**lettern **u**nd **S**timmung’; ‘Climbing and Mood’) was to investigate the effectiveness of a manualised bouldering psychotherapy (BPT), compared with exercise alone, in a large nationwide sample of outpatients with depression. For this purpose, we used an active control group, involving a home-based exercise programme (EP), instead of a waitlist control group. The home-based exercise programme was designed to be an externally supervised opportunity to engage in exercise such as often offered by health insurance providers. We hypothesised that participation at BPT would lead to a significantly greater reduction in depressive symptoms than mere physical activation in the form of the EP. The effect of BPT on other measures of mental health (e.g. self-esteem, coping skills, anxiety) was examined with an exploratory approach.

## Methods

### Study design

**Fig. 1:**
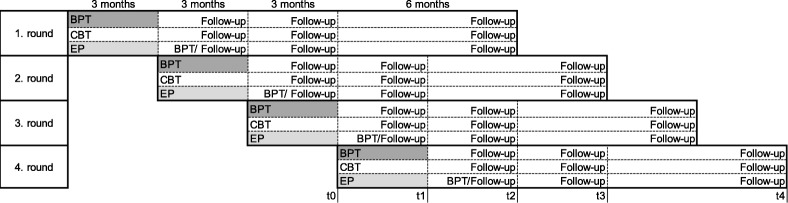
Study Design. *Notes.* BPT: Bouldering psychotherapy; CBT: cognitive behavioural therapy; EP: exercise programme; Erlangen and Weyarn/Munich: four waves, Berlin: two waves; measurement occasions are shown by using the fourth wave as an example

The study design was already described in detail in our study protocol and the following descriptions are based on the explanations there [37]. The StudyKuS began in 2016 as a multicentre, randomised, controlled study. It was conducted in three different regions across Germany: a) Erlangen/Nuremberg/Fuerth (metropolitan region), b) Weyarn (rural region), and c) Berlin (capital region). Within one region, the three interventions offered in the study (BPT, CBT or EP) took place during the same time period and were conducted in consecutive waves, with four waves in the Erlangen/Nuremberg/Fuerth region as well as the Weyarn region and two waves in the Berlin region. Participants within one region and one wave were randomly allocated to one of three groups (BPT, CBT, or EP). Subsequent to the therapy period, participants, who were assigned to the EP were offered the opportunity to participate in an additional ten-week bouldering group, which followed the same treatment plan as the BPT group and took place directly after the therapy period (see Fig. 1). Data were collected via computer-assisted telephone interviews (CATIs) before start of the therapy (t0), at the end of the therapy (t1), and 3 months (t2), 6 months (t3), and 12 months (t4) after the therapy period (Follow-Up). For details of the data collection please see our study protocol [37]. Interviewers conducting the CATIs were blinded with respect to participants’ allocations. Participation was voluntary, and participants were free to leave the study at any time. All procedures were approved by the Friedrich-Alexander Universität of Erlangen-Nürnberg Ethics Committee (Ref. 360_16 B).

### Interventions

#### Bouldering psychotherapy (BPT)

Our newly developed bouldering psychotherapy is a combination of bouldering and psychotherapy. The programme consists of ten consecutive sessions of 2 hours. In the current study, it took place in groups of about ten participants in a bouldering gym once a week in the late afternoon. In each study centre, therapeutic teams consisted of two therapists, but the personnel composition varied across the different waves of therapy because the therapists sometimes had other commitments (a total of nine climbing therapists). For qualification of the therapists see the study protocol [37]. Each of the ten BPT sessions focussed on a specific psychological topic that was considered to be relevant in the development and maintenance of depression. Table 1 shows an overview of the specific subjects covered in the ten therapeutic sessions [see also 37]. Each session was based on the BPT manual and followed a standardised procedure (introduction, action phase, closure), comprising mindfulness exercises, psychoeducational elements, topic-related bouldering exercises under therapeutic supervision, exchange of individual experiences between participants and transfer to daily life, body-related relaxation exercises, and free bouldering. The purpose of the bouldering exercises was to evoke underlying emotions (e.g. anxiety), unveil patients’ characteristic patterns (e.g. avoidance), and enable patients to engage in new experiences (e.g. exposition: bouldering blindfolded). For a detailed description of the treatment, see the study protocol [37].

**Table 1:** Overview of sessions of the BPT [37]

Session	Topic
1	Introduction to bouldering and mindfulness
2	Physical feeling and body’s centre of gravity
3	Healthy handling of limitations
4	Expectations and standards
5	Self-efficacy, achievements, and pride
6	Self-esteem
7	Fear and trust I
8	Fear and trust II
9	Social relationships
10	Problem solving, reflecting on lessons learned, and transfer to daily life

#### Exercise programme (EP)

The home-based Exercise Programme was supposed to address the same muscles as used in bouldering. It consisted of a 20-min physical training programme that was conducted by the participants on their own at home, using a training DVD and/or a training manual (with instructions and explanations for all of the exercises). Additionally, participants received training material (e.g. a multifunctional latex band and training rings to enhance finger and arm power) as well as psychoeducational material explaining the positive effect of physical exercise on mood. Participants were instructed to perform the exercises about three times a week for ten weeks (resulting in 60 min per week, comparable to the ‘active time’ in the BPT group). At regular intervals, they received reminders and motivational material to keep on exercising. Depending on their personal preferences, participants were contacted via e-mail, telephone, or postal mail. As opposed to other Internet-based offers of therapy (i.e. by health insurance companies), people without Internet access or an e-mail address were also able to participate at the EP group. In addition, participants were provided with an exercise diary that encouraged them to record their training sessions and subsequently rate their mood. After the intervention period, the total rate of exercise was assessed in a personal interview with an external rater via self-report.

#### Cognitive behavioural therapy (CBT)

The third intervention that was applied was based on a classical cognitive behavioural group therapy but will not be described in detail because it is not relevant to the hypotheses we tested in this article. For a detailed description of the treatment, see the study protocol [37] and other publications by the work group.

### Recruitment and randomisation

Between January 2017 and March 2018, participants were recruited by the distribution of informational materials (e.g. flyers, posters) in relevant institutions (e.g. psychiatric hospitals, psychotherapists’ offices, primary care physicians’ offices, pharmacies, support groups). Additionally presentations were held at local events and press releases were issued and addressed to different local newspapers and radio stations prior to the start of each intervention wave. A homepage (www.studieKuS.de) and a Facebook account were created and regularly updated. Informational sessions were held by the study personnel, in which all people interested in the study were informed about the conditions surrounding a participation in the study (e.g. randomisation). Those willing to participate were asked to fill out a short screening questionnaire and to declare their written informed consent. All individuals fulfilling the inclusion criteria were informed that they had been included in the study and subsequently randomised blockwise within one region and one wave to one of the three groups (BPT, CBT, EP). Randomisation was stratified by sex and severity of depression (PHQ-9 scores 8–14 mild, 15–19 moderate, 20–27 severe depression). Randomisation was performed by the Institute of Medical Informatics, Biometrics, and Epidemiology (IMBE) at the Friedrich-Alexander-Universität Erlangen-Nürnberg with a computer-based system and was based on participants’ codes without the statistician’s knowledge of assignment to the intervention arms.

### Inclusion and exclusion criteria

Eligibility was determined through the screening questionnaire handed out at the information sessions or upon request. Only a few inclusion and exclusion criteria were applied, in order to increase the external validity of the study. Potential participants were personally interviewed by the study personnel if the fulfilment of the inclusion or exclusion criteria was unclear. *Inclusion criteria* were acute depressive symptoms, informed consent to participate in the study (especially approval for randomised allocation and data collection), and the ability to get to the therapy locations. The presence (or absence) of depression was operationalised by a PHQ-9 score of at least eight points, ensuring a high level of sensitivity for all depressive disorders [38]. *Exclusion criteria* were an age under 18 years, a Body Mass index (BMI) under 17.5 or over 40, contemporary participation in another psychotherapeutic group therapy, started psychiatric medication or psychotherapy within the last 8 weeks, planned inpatient stay during the intervention period, physical contraindication for bouldering (physical disorders or pregnancy), specific psychiatric disorders (psychosis within the last 5 years, a manic episode within the last 5 years, substance addiction with substance abuse within the last year, borderline personality disorder diagnosis with self-harming behaviour during the last year), and acute suicidality [see also 37]. All participants were obliged to sign an anti-suicide contract for the duration of the study. After randomisation, participants were informed about their allocation and provided with all the necessary information about group participation.

### Instruments

#### Primary outcome measure

*Montgomery–Åsberg Depression Rating Scale (MADRS)* [39]. The MADRS is one of the most commonly used rating scales for assessing core symptoms of depression [40]. It consists of ten items, which are rated by a clinician in a semi-structured interview on a seven-point scale with higher scores indicating greater severity of depression (ten or below: remission, greater than 31: severe depression) [41]. In our study, the *structured interview guide for the Montgomery–Åsberg Depression Rating Scale (SIGMA)* [40] was used, which offers a selection of different questions for each item.

#### Secondary outcome measures

Subscale *interpersonal sensitivity* of the *Symptom-Checklist (SCL-90)* [42, 43]. The SCL-90 is a self-report inventory which measures among other variables the intensity of distress experienced during the past 7 days caused by interpersonal sensitivity. Ratings on the five-point Likert-type scale were summed, and standardised t-values were computed, with higher scores indicating an increasing severity of symptoms.

*Generalised Anxiety Disorder 7 (GAD-7)* [44, 45]. The GAD-7 is a brief self-report questionnaire, asking patients how often they have felt bothered during the last two weeks by each of the seven core symptoms of generalised anxiety disorder. Items can be rated on a four-point scale, with higher sum scores indicating higher anxiety (≥ 5 mild ≥10 moderate, and ≥ 15 severe anxiety).

Subscale *vital body dynamics* of the *Body Image Questionnaire (Fragebogen zum Körperbild, FKB-20)* [46]. The FKB-20 assesses body image disturbances and subjective aspects of body experience. The vital body dynamics subscale consists of ten items rated on a five-point scale with higher sum scores indicating a more positive body image.

Subscale c*oping* of the *Questionnaire on Resources and Self-Management Skills (Fragebogen zur Erfassung von Ressourcen und Selbstmanagementfähigkeiten, FERUS)* [47]. The FERUS assesses an individual’s health-related resources and manageability, among others on the subscale *coping* [48]. Twelve items are rated on a five-point Likert scale. Ratings were summed with higher test scores indicating better resources and manageability skills.

Rosenberg Self-Esteem Scale (R-SES) [49]. The R-SES is a self-report instrument for evaluating global self-worth. It consists of ten items answered on a four-point scale with higher values indicating higher self-esteem.

As an additional measure for depression the 9-Item *Patient Health Questionnaire (PHQ-9)* [44, 50] was applied*.* The PHQ-9 is a short self-assessment tool which is often used for the screening of depression in primary care settings [51]. Its nine items cover the nine DSM-IV criteria and are rated on a four-point scale. The total sum score suggests varying levels of depression (0–4 minimal depression, 5–9 mild depression, 10–14 moderate depression, 15–19 moderately severe depression, 20–27 severe depression) [38, 50].

For all of the psychometric measures, change scores were computed as the difference between t1 and t0.

#### Other outcome measures

Other variables that were assessed either through the screening questionnaires or via the CATIs included, among others, sociodemographic data (e.g. age, gender, and level of education), body mass index (BMI), current therapeutic treatment (antidepressant medication, additional psychotherapy), psychiatric comorbidities, medical history of depression, critical life events, physical limitations, and attitude towards physical activity (positive or negative). The interview guide regarding those variables is presented in Additional file 1.

For a comprehensive depiction of all the measures we assessed, see the study protocol [37].

### Statistical analysis

To increase the power of the statistical procedures, all participants in each group (BPT and EP) were combined across the three study centres and two/four waves for the main analyses. Descriptive statistics (frequencies, means, and standard deviations) were computed to illustrate sample and baseline characteristics. To assess the quality of the randomisation, differences between the EP and BPT groups in the variables of interest were evaluated via two-sample t-tests, *U*-tests, chi-square (*χ*2) tests, and univariate analyses of variance (ANOVAs). The underlying assumptions of parametric tests were checked before using the Kolmogorov-Smirnov and Levene’s test. Baseline variables that were significantly different between the two groups were included as confounders in the multiple regression model. All data were checked for plausibility. A missing data evaluation was carried out, and missing values were imputed using the expectation maximisation (EM) algorithm. The primary data analysis strategy was ‘per protocol’ (PP). Participants who dropped out during the intervention period were subsequently interviewed and included in the ‘intention to treat’ (ITT) analyses. Dropout analyses were computed to check for differences between participants who dropped out and those who completed the study, using χ^2^ tests, *U*-tests and two-sample t-tests.

To check for pre-post changes within the groups in the main outcome criterion (i.e. depressive symptoms assessed with the MADRS), paired t-tests were computed to compare the changes between t0 and t1 in both the BPT and EP group. To compare the improvements between the two groups, t-tests for independent groups were computed to compare the change scores (t1-t0) between the BPT and EP group. In addition, a multiple regression analysis was calculated to predict the post-intervention (t1) MADRS score from group allocation (BPT vs. EP), controlling for demographic variables (age, sex), participants’ BMI, attitude towards sports, other therapeutic treatments (antidepressant medication, additional psychotherapy), and severity of depression at baseline (MADRS t0 score).

As a sensitivity analysis, additional analyses with ITT data were computed and compared with the results of the PP analyses. Furthermore, an additional regression analysis controlling for the study centre was calculated to rule out potential centre effects. Collinearity statistics were examined in advance to ensure there were no issues with multicollinearity.

Secondary outcomes as well as depression measured by the PHQ-9 were tested in an exploratory fashion. To check for improvements within the groups, paired t-tests were computed to assess changes between t0 and t1 for the EP and BPT. After checking for homogeneity of variance, change scores were compared with t-tests for independent groups and *U*-tests (as sensitivity analyses) between the two groups. As a measure of effect size, Cohen’s *d* was calculated.

To determine consistency among the raters, for 10 (4%) of the pre-intervention (t0) and 11 (5%) of the post-intervention (t1) CATIs, a second person also rated the interviewee’s answers on the SIGMA and intraclass correlations (ICCs) were computed across all groups to assess interrater reliability. For all analyses, a Type 1 error rate (alpha) of less than 5% was considered to indicate statistical significance. Statistical analyses were performed with the aid of the IBM SPSS Statistics 21 software.

## Results

### Description of study participants

**Fig. 2:**
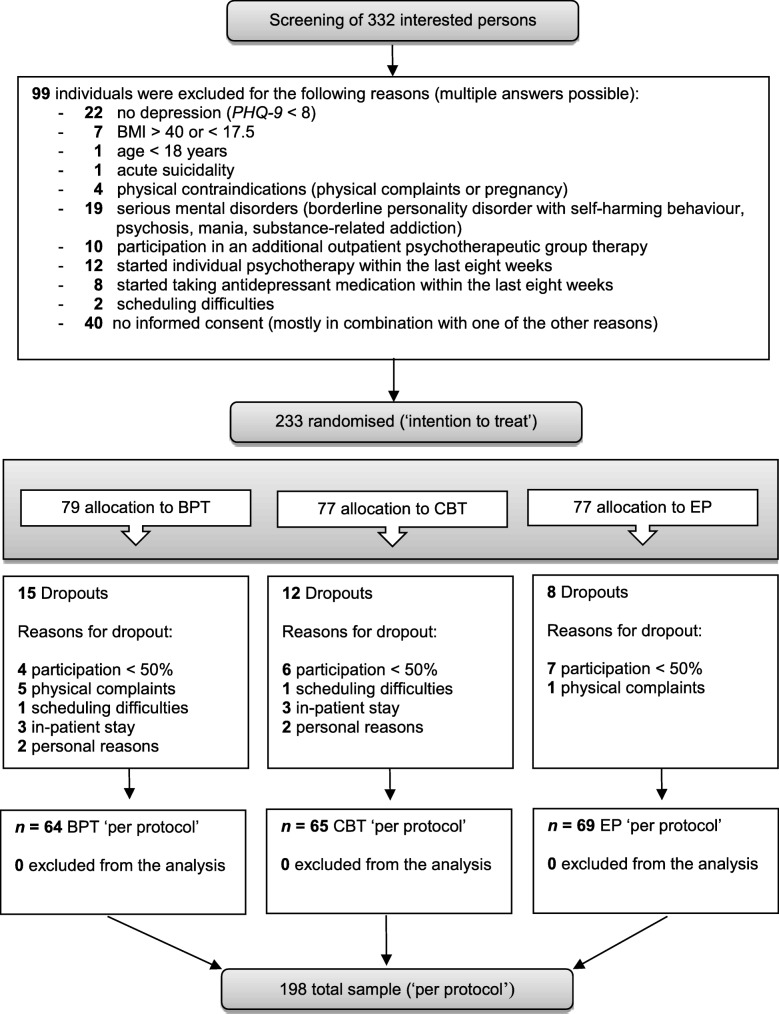
Consort Flow Chart. *Notes.* BPT: Bouldering psychotherapy, CBT: cognitive behavioural therapy, EP: exercise programme

The intervention period ran from May 2017 to June 2018. Of 332 individuals who attended the screening, 99 of them did not meet the inclusion criteria (see Fig. 2 for the reasons for exclusion). A total of 233 participants were included in the study (ITT). 79 participants were randomly assigned to the BPT, 77 to the CBT, and 77 to the EP group. Figure 2 shows the flow of participants through the study. During the course of the therapies, 35 participants either dropped out of the study (for their reasons, see Fig. 2) or participated in fewer than five sessions (50%) in their allocated group. The per protocol sample consisted of 198 participants (BPT: *n = 64*, CBT: *n = 65*, EP: *n = 69;* PP). For the analyses that we computed to investigate our specific research question, only participants in the BPT and EP groups (PP: *n = 133*) were included. All subsequent descriptions pertain to this subsample. Analyses involving the CBT group will be part of further investigations.

The sample characteristics and group differences between the BPT and EP groups are shown in Table 2.

**Table 2:** Sample Characteristics

Variable	BPT (*n* = 64)		EP (*n* = 69)		Total (*n* = 133)		Test of group differences		
							*F*/χ^2^	*T/ U*	*p*
Age ^a^, *M* (*SD*)	41.0	(12.2)	42.8	(12.7)	42.0	(12.5)		2025.00	.410
Sex: female, *n* (%)	45	(70.3)	47	(68.1)	92	(69.2)	0.08		.784
School education, *n* (%)							0.36		.550
< 9 years	0	(0.0)	1	(1.4)	1	(0.8)			
9 years	6	(9.4)	4	(5.8)	10	(7.5)			
10 years	16	(25.0)	24	(34.8)	40	(30.1)			
≥ 12 years	42	(65.6)	40	(58.0)	82	(61.7)			
BMI ^a^, *M* (*SD*)	23.8	(3.3)	24.8	(4.3)	24.3	(3.9)		1883.50	.144
Attitude towards sports: positive, *n* (%)	62	(96.9)	68	(98.6)	130	(97.7)	0.42		.516
Additional psychotherapy: yes, *n* (%)	38	(59.4)	24	(34.8)	62	(46.6)	8.07		.005*
Antidepressants: yes, *n* (%)	32	(50.0)	33	(47.8)	65	(48.9)	0.06		.802
First depressive episode: yes, *n* (%)	15	(23.4)	21	(30.4)	36	(27.1)	0.82		.364
Number of depressive episodes ^b^, *n* (%)							0.01		.941
1–2	5	(8.9)	10	(18.2)	15	(13.5)			
3–4	14	(25.0)	5	(9.1)	19	(17.1)			
5–10	6	(10.7)	9	(16.4)	15	(13.5)			
< 10 or chronic depression (> 2 years)	31	(55.4)	31	(56.4)	62	(55.9)			
Additional morbidities ^c^, *MDN* (*IQR*)	0	(1)	0	(1)	0	(1)		2161.50	.812
Baseline measures (t0)									
Depression									
MADRS, *M* (*SD*)	23.5	(8.9)	22.2	(9.1)	22.8	(9.0)		−0.81	.421
PHQ-9, *M* (*SD*)	13.5	(5.5)	13.2	(5.0)	13.4	(5.2)		−0.31	.756
Anxiety (GAD-7), *M* (*SD*)	11.0	(4.2)	11.3	(4.2)	11.1	(4.2)		0.50	.618
Body Image (FKB-20), *M* (*SD*)	24.0	(6.7)	23.3	(6.2)	23.7	(6.4)		0.63	.533
Global self-esteem (R-SES) ^a^, *M* (*SD*)	13.3	(5.6)	14.2	(5.1)	13.8	(5.4)		1961.50	.266
Coping (FERUS), *M* (*SD*)	33.2	(7.5)	33.9	(6.8)	33.6	(7.1)		0.59	.558
Interpersonal sensitivity (SCL-90) ^a^, *M* (*SD*)	68.9	(8.6)	69.0	(7.5)	68.9	(8.0)		2208.00	1.00

*Note. BMI* Body Mass Index; *MADRS* Montgomery and Åsberg Depression Rating Scale; *PHQ-9* Patient Health Questionnaire 9 Items; *GAD-7* Generalised Anxiety Disorder 7; *FKB-20* Body Image Questionnaire; *R-SES* Rosenberg Self-Esteem Scale; *FERUS* Questionnaire on Resources and Self-Management Skills; *SCL-90* Symptom-Checklist; *MADRS* scores > 31 indicate severe depression, scores ≤10 indicate remission; *PHQ-9* 0–4: minimal depression, 5–9: mild depression, 10–14: moderate depression, 15–19: moderately severe depression, 20–27: severe depression; *GAD-7* scores ≥5, ≥ 10, and ≥ 15 indicate mild, moderate, and severe anxiety symptoms

^a^ the Kolmogorov-Smirnov test indicated a significant deviation from a normal distribution, but no deviation was observed via histograms; therefore, the mean value (*M*) and standard deviation (*SD*) is reported; ^b^*n* = 211 (22 missing values); ^c^ both the Kolmogorov-Smirnov test and an optical inspection (histogram) indicated a significant deviation from a normal distribution; therefore, the median (*MDN*) and the interquartile range (*IQR*) are reported; * *p* ≤ .05

Of the 133 participants remaining after the 10 weeks (PP), 69.2% were female and 31.8% were male. The mean age of participants was 42 years (*SD* = 12.5), the average weight fell within the normal range (BMI: *M* = 24.3, *SD* = 3.9), and the majority (61.7%) had completed 12 years of schooling. Almost all of the participants (97.7%) reported a positive attitude towards sports. Across the groups, around half of the participants (46.6%) underwent additional outpatient psychotherapy and received antidepressant medication (48.9%) in addition to participating in the study. More than two thirds of the participants (72.9%) reported at least one depressive episode before the current one, and more than half (55.9%) reported at least ten depressive episodes in the past or claimed to be suffering from chronic depression (the current episode had been running for more than 2 years). The average depression score at the beginning of the study indicated a moderate level of depression (PHQ-9: *M* = 13.4, *SD* = 5.2; MADRS: *M* = 22.8, *SD* = 9.0). Other baseline measures are presented in Table 2. Overall, the groups were comparable with respect to the baseline scores of the psychometric measures and key characteristics, with the exception of additional psychotherapeutic treatment: Participants in the EP group received additional psychotherapy less often (*n* = 24, 34.8%) than the BPT group (*n* = 38, 59.4%). Over the 10 weeks, participants in the BPT group took part in eight BPT sessions (*M* = 7.91, *SD* = 1.48), and participants in the EP group engaged in 2.2 sessions of the exercise programme per week (in total: *M* = 21.90, *SD* = 14.04). Participants who dropped out between t0 and t1 (*n = 23*) did not differ from the remaining sample in age, sex, BMI, additional psychotherapeutic or antidepressant treatment received, or severity of depression (PHQ-9 and MADRS scores at t0). The interrater reliability for the main outcome, the SIGMA, was excellent (*ICC* = .985, 95% *CI* from .963 to .994, *p* < .001).

### Main outcome

#### Univariate results

**Fig. 3:**
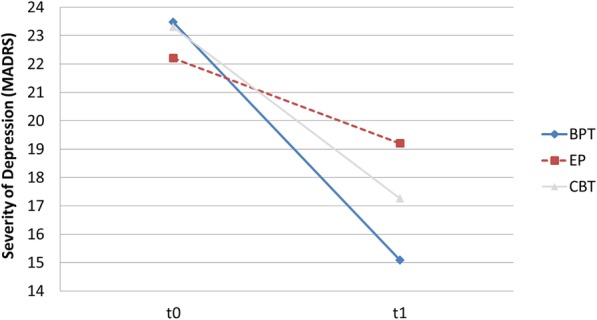
Change in Depression Scores between t0 and t1 on the MADRS. *Notes.* BPT: Bouldering psychotherapy, CBT: cognitive behavioural therapy, EP: exercise programme

Both groups indicated significant improvements in depressive symptoms after the ten-week intervention period. In the BPT group, depression scores dropped by 8.4 points on the MADRS (t0 = 23.5 vs. t1 = 15.1; *p* = .003) over the course of the ten-week intervention period, whereas the EP group’s scores dropped by only 3.0 points on the MADRS (t0: 22.2 vs. t1: 19.2; *p* < .001) during the same time period (BPT vs. EP: *p* = .002) (see Fig. 3). On average, participants in the BPT group improved by more than one severity grade, from moderate to mild depression (MADRS score ≤ 19), while the improvement of the EP group remained within the same severity grade (moderate depression). The effect size (intervention vs. active control group) was moderate (Cohen’s *d* = 0.55) (see Table 3). The sensitivity analysis with ITT data showed comparable results between groups (BPT: 8.1 vs. EP: 3.0, *p* = .001, Cohen’s *d* = 0.53). For a better interpretation of the results, we included descriptive results of the third intervention arm, the CBT group, in Fig. 3.

**Table 3:** Group Differences between the BPT and the EP Group for Change Scores between t0 and t1

	BPT (*n* = 64)	EP (*n* = 69)	Cohen’s *d*	*Independent t*-test	*U*-test
Scale	*∆M* (*SD*)	*∆M* (*SD*)		*p*	*p*
Primary outcome					
Depression (MADRS)	−8.4 (10.4)^+^	−3.0 (9.3)^+^	0.55	.002*	.001*
Secondary outcomes					
Anxiety (GAD-7)	−3.4 (4.3)^+^	−2.0 (4.0)^+^	0.35	.046*	.079^†^
Body Image (FKB-20)	3.5 (6.6)^+^	1.0 (5.3)	0.42	.018*	.010*
Global self-esteem (R-SES)	3.6 (4.2)^+^	1.7 (4.3)^+^	0.45	.011*	.007*
Active and passive coping (FERUS)	3.2 (7.2)^+^	1.3 (6.4)	0.28	.115	.130
Interpersonal sensitivity (SCL-90)	−3.5 (6.2)^+^	−3.0 (5.8)^+^	0.09	.591	.880
Additional measure					
Depression (PHQ-9)	−4.7 (6.3)^+^	−2.6 (5.2)^+^	0.36	.041*	.043*

*Note.* Differences between t0 and t1 (t1-t0): Negative values on the MADRS, PHQ-9, GAD-7, and SCL-90 indicate improvements in symptoms, positive values on the FKB-20, GSE, R-SES, and FERUS indicate positive effects

^+^ indicates a significant difference between t0 and t1 within the group; ^†^*p* ≤ .10; * *p* ≤ .05

#### Regression analysis

Results of the confounder-adjusted regression analysis (see Table 4) revealed a significant effect of group allocation (BPT vs. EP) on the depression score after the intervention period (MADRS at t1) with BPT participants showing significantly lower depressive symptoms than participants in the EP (*β* = − 5.60, *p* = .001). In addition, the baseline depression score (MADRS at t0) emerged as a significant predictor (*β* = 0.55, *p* < .001). No significant effects were found for any of the other control variables. The sensitivity analysis with ITT data showed similar results (group allocation: *β* = − 5.12, *p* = .001; MADRS t0: *β* = 0.55, *p* < .001) and no significant study centre effects were revealed (Berlin vs. Erlangen: *β* = 0.613, *p* = .739; Weyarn vs. Erlangen: *β* = 2.483, *p* = .185).

**Table 4:** Summary of the Multiple Regression Analysis for Variables Predicting Depression (MADRS) at t1

Predictor	*β*	*p*	95% CI	
			*LL*	*UL*
Group allocation: BPT	−5.601	.001*	*−8.864*	*−2.338*
t0 MADRS sum score	.545	< .001*	*.367*	*.724*
Sex: female	1.507	.421	*−2.184*	*5.198*
Age	−.048	.479	*−.182*	*.086*
BMI	−.144	.503	*−.569*	*.281*
Attitude towards sports: positive	−3.252	.542	*−13.781*	*7.277*
Antidepressants: yes	.805	.624	*−2.440*	*4.051*
Psychotherapy: yes	1.823	.279	*−1.493*	*5.139*

*Note*. *MADRS* Montgomery and Åsberg Depression Rating Scale, higher scores indicate more severe symptoms; *CI* confidence interval, *LL* lower limit, *UL* upper limit, *β* unstandardized regression coefficient

* *p* ≤ .05

### Secondary outcomes

During the ten-week intervention period, participants in the BPT group showed significant positive changes in anxiety, body image, active and passive coping, global self-esteem and interpersonal sensitivity (see Table 3). For the EP, significant differences between t0 and t1 were observed for the same variables with the exception of body image and coping. However, pre-post changes were significantly higher within the BPT group compared with the EP group for anxiety (GAD-7: *p* = .046, *d* = 0.35), body image (FKB-20: *p* = .018, *d* = 0.42), and global self-esteem (R-SES: *p* = .011, *d* = 0.45) (see Table 3).

Results of the additional measure on depression, the PHQ-9, support the findings of the MADRS: Scores dropped by 4.7 points (t0 = 13.5 vs. t1 = 8.9; *p* = .003) in the BPT group and only by 2.6 points in the EP group (t0: 13.2 vs. t1: 10.6; *p* < .001) during the same time period (BPT vs. EP: *p* = .041).

## Discussion

To our knowledge, this is the first randomised controlled intervention trial to compare the benefits of a therapeutic bouldering intervention to that of a home-based physical exercise programme in outpatients with depression. As we expected, depressive symptoms were effectively reduced in both the bouldering therapy group and the exercise group during the ten-week intervention period. These results are in accordance with previous studies on the antidepressant effects of physical exercise [8] and also parallel previous findings by our work group on the benefits of psychotherapeutic bouldering in treating depressive symptoms [35, 36]. What has not been shown before and therefore represents new insights into the relation between physical activity and depression is the finding that the effect of the BPT seems to go even further than the effect of only physical activity. Comparing the change scores in the two groups, the benefits of therapeutic climbing significantly exceeded those of the exercise programme. While the scores of the participants in the BPT group dropped by 8.4 points on the MADRS, thus constituting an average improvement from moderate to mild depression, the scores of the participants in the EP group dropped by only 3.0 points on the MADRS and thereby remained within the range of moderate depression. Simultaneously, in the confounder-adjusted regression analysis, group allocation (BPT vs. EP) emerged as the only relevant predictor of the post-intervention depression score measured with the MADRS. These findings underpin and augment the previous results of our work group’s pilot trial [36], which suggested therapeutic effects of a bouldering psychotherapy intervention on depressive symptoms even when general activity was controlled for. With a moderate effect size (Cohen’s *d*) of 0.55 for the MADRS, the effect of the BPT intervention applied in the current study was weaker than that of the BPT in the pilot study (Cohen’s *d* = 0.77). However, one important factor contributing to the less pronounced effect size is that in the present study, an active control group in the form of an exercise programme was used instead of an untreated waitlist control group as applied in the pilot study. Participants in the EP knew that they could participate in an additional subsequent BPT after they completed the EP in 10 weeks. Hope has been shown to be inversely correlated with depressive symptoms [52], hence we assume that the antidepressant effect of the EP was further increased by the expectation of an improvement through participation in the subsequent BPT group. Therefore, the effect of the EP is likely to be overestimated in our study.

Still, the moderate effect is comparable to the antidepressant effect of other short-term group therapies [53] or physical exercise [6, 54–59]. In an exploratory approach, additional favourable effects of the EP as well as BPT were found for additional secondary mental health outcomes apart from depression. While participants in both groups showed improvements in anxiety, global self-esteem and interpersonal sensitivity after the ten-week intervention period, those in the BPT group even improved in body image and active and passive coping. As already observed for the primary outcome, significantly higher pre-post changes in anxiety, body image, and global self-esteem were revealed for the BPT compared with the EP, which again indicates the superiority of therapeutic bouldering to physical exercise alone. In summary, physical activity has been reconfirmed to play an important part in alleviating depressive symptoms. However, it seems to represent only one of several therapeutic factors through which our bouldering psychotherapy exerts its antidepressant effect.

### Strengths and limitations of the study

#### Strengths

The StudyKuS is the first controlled intervention trial to compare the effectiveness of a manualised bouldering psychotherapy (BPT) with an active control group (EP) in a large nationwide sample of individuals suffering from depression. The randomised controlled but still naturalistic design involved only a few inclusion and exclusion criteria to maximise external validity and thus to allow for the generalisability of the findings. By randomly allocating participants to either the BPT group or the EP group, relevant confounding factors were controlled for, and a high level of internal validity was achieved. Depressiveness as the primary outcome was assessed via the MADRS, a clinician-rated interview, and we were thereby able to avoid the potential biases that are often associated with self-report questionnaires. To obtain a maximum of standardisation, interviewers followed a structured interview guide, which offered a selection of different questions for each of the MADRS items. Due to the longitudinal design of the study with a long follow-up period of 1 year, potential long-term effects of BPT as well as the EP can be investigated in future studies.

#### Limitations

Despite randomisation, a significant between-group difference in additional outpatient psychotherapy emerged at baseline in the direction that participants in the EP less often received additional psychotherapy than those in the BPT group. In order to rule out any bias in the results in favour of the BPT intervention, additional psychotherapy at baseline was controlled for in the multiple regression analysis and was found to have no significant effect on the post-intervention depressiveness score. Participants in the EP group were instructed to engage in the exercises given in the EP at least three times a week, and the number of sessions completed was assessed via self-report after the intervention period. However, there was no external control of the duration of each session or whether the training units were completed at all. In future studies, a more reliable assessment of the total amount of time spent exercising would be beneficial. With regard to the setting, it should be borne in mind that the BPT was conducted as a group intervention, and therefore, psychoeducation was provided by a therapist, whereas the EP was carried out alone at home, and psychoeducation was thus provided by a brochure. As the feeling of belonging to a group, called group cohesiveness, constitutes a therapeutic impact factor in the treatment of mental disorders [60], it is difficult to rule out the effect of the group when comparing the two interventions. An additional effect in favour of the BPT might have resulted from higher expectations of the BPT intervention as participants had specifically applied for a study on “bouldering”. A benefit of the EP, as already mentioned before, was that the participants knew that they would be able to participate in a bouldering group after they completed the EP.

#### Future research perspectives

Regarding future research on this topic, a direct comparison between BPT and a regular non-psychotherapeutic bouldering group as well as other modes of group exercise (i.e. running groups) could help to provide insight into the underlying mechanisms. To go a step further, it would be of great interest to compare the effectiveness of BPT with established and guideline-recommended approaches for the treatment of depression such as cognitive behavioural therapy. Also, in order to draw reliable conclusions about the long-term effects of BPT, follow-up data (up to 1 year after the intervention) must be evaluated. Both questions will be dealt with by our work group. Finally, further studies involving specific target groups (i.e. grouped by sex, specific ages, participants’ sportiness) and different mental disorders (i.e. somatic symptom disorder, substance addiction) would be beneficial in order to allow a selective, more targeted implementation of BPT.

## Conclusions

The results of the current study provide support for previous findings in suggesting positive effects of physical activity and particularly bouldering in depressed individuals. Moreover, it is evident that our bouldering psychotherapy is not only efficacious in reducing depressive symptoms but even goes beyond the benefits of mere physical exercise. There is good reason to believe that psychotherapeutic bouldering may be a viable alternative to classical mental health treatments, particularly when addressing patients whose needs are not covered by the current care system. Future research on the efficacy of our bouldering psychotherapy on relevant subgroups and other mental disorders would be beneficial in order to allow a more targeted implementation of BPT in clinical practice.

## Supplementary information

**Additional file 1.** Interview guide - questions raised during CATIs in addition to the validated instruments.

## Data Availability

All the results supporting our conclusions are contained in the manuscript. The datasets that were used and/or analysed in the current study are available from the corresponding author upon reasonable request after the publication of the results.

## Abbreviations

BMI: Body Mass Index
BPT: Bouldering Psychotherapy
CATI: Computer-Assisted Telephone Interview
CBT: Cognitive Behavioural Therapy
EP: Home-Based Exercise Programme
FERUS: Questionnaire on Resources and Self-Management Skills
FKB-20: Body Image Questionnaire
GAD-7: Generalised Anxiety Disorder 7
IQR: Interquartile Range
M: Mean
MADRS: Montgomery–Åsberg Depression Rating Scale
MDN: Median
PHQ-9: Patient Health Questionnaire 9 Items
R-SES: Rosenberg Self-Esteem Scale
SCL-90: Symptom-Checklist
SD: Standard Deviation
StudyKuS: Study ‘Climbing and Mood’
