# Supplementary material for: Bouldering psychotherapy is more effective in the treatment of depression than physical exercise alone: results of a multicentre randomised controlled intervention study
Source: BMC Psychiatry. 2020 Mar 12;20:116. doi: 10.1186/s12888-020-02518-y (PMC7066840; doi:10.1186/s12888-020-02518-y)
Supplement: Supplementary file 1 — Additional file 1. Interview guide - questions raised during CATIs in addition to the validated instruments. [file 12888_2020_2518_MOESM1_ESM.pdf]

Interview Guide

1. General questions

1.1 Greeting

For the interviewer: Please find your own words!

- Introduction. You have decided to participate in study KuS. Before the real start we would like to conduct our first telephone interview with you today.
- The telephone conversation will last about one hour; data will be treated in strict confidence.
- It may be easier for you if you have the questionnaire in front of you.
- It is important that we as interviewers do not know which group you have been assigned to. Therefore, if possible, do not tell us whether you are in the bouldering, depression or exercise group.

1.2 Date of birth: ______________________________

1.3 Gender

For the interviewer: please fill in independently

☐ female

☐ male

1.4 Family status

☐ single

☐ married/ living in a partnership

☐ segregated

☐ divorced

☐ widowed

1.5 Do underage children life in your household?

☐ yes

☐ no

1.6 What is your highest school or university degree?

☐ no school degree

☐ “Hauptschule”/ elementary school

☐ “Realschule”/ secondary school

☐ “Abitur”/ High school graduation

1.7 Are you currently employed?

☐ yes

☐ no

☐ never been

If YES: you are currently employed

1.7.1 What is your professional position?

☐ *self-employed*

☐ *employed*

☐ *official*

1.7.2 How many hours/week do you work in total?

For the interviewer: this means the contractually agreed working time (even if on sick leave)
Total working time: _______ hours per week

1.7.3 Do you currently work with reduced hours due to your mental illness?

☐ *yes, reduced by _____________ hours per week*

☐ *no*

1.7.4 Are you currently on sick leave due to your mental illness?

☐ *yes, since ____________ (month/year) until about ____________ (month/year)*

☐ *no*

1.7.5 Have you been on sick leave in the last six months due to your mental illness?

☐ *yes, about ______________ weeks*

☐ *no*

1.7.6 Please indicate how often you have been restricted in your professional activities in the last month as a result of your psychological well-being.

☐ *Missing an entire working day: ________ times*

OR ☐ *Missed working hours per month: about ________ hours*

If NO: you are not employed

1.7.7 Why did you stop working??

☐ *retirement age reached*

☐ *prematurely retired (not due to depression)*

☐ *termination*

☐ *children*

☐ *on account of mental state*

☐ *miscellaneous:* ___________________________

1.7.8 When did you stop working?

Since ______________ *(month/year)*

1.8 Has there been a serious negative event in your life in the last 2 weeks?

☐ yes

☐ no

If YES: there has been a serious negative event

1.8.1 Which? ____

2. Questions about your illness and psychotherapeutic treatment

2.1 Are you currently suffering from depression <u>for the first time</u>?

☐ yes

☐ no

If YES: you are currently suffering from depression for the first time

2.1.1 Since when do you suffer from depression?

Since about ____________ *(month/year)*

If NO: you are not suffering from depression for the first time

2.1.2 How many depressive phases have you had?

____________ *phases*

2.1.3 When did the current depressive phase start?

About ____________ *(month/year)*

2.2 Are you taking medication regularly because of your mental state?

☐ yes

☐ no

If YES: you are taking medication regularly

2.2.1 Which medication, in what dose and since when?

☐ *Amitriptylin, dose (mg): ___________, since ________ month*

☐ *Citalopram, dose (mg): ___________, since ________ month*

☐ *Duloxetin, dose (mg): ___________, since ________ month*

☐ *Escitalopram, dose (mg): ___________, since ________ month*

☐ *Fluoxetin, dose (mg): ___________, since ________ month*

☐ *Mirtazapin, dose (mg): ___________, since ________ month*

☐ *Opipramol, dose (mg): ___________, since ________ month*

☐ *Sertralin, dose (mg): ___________, since ________ month*

☐ *Venlafaxin, dose (mg): ___________, since ________ month*

☐ *Miscellaneous: ____________________________________,*

dose (mg): ___________, since ________ month

2.3 Are you currently undergoing outpatient psychotherapeutic treatment?

For the interviewer: ask exactly! No quarterly contacts with the doctor!

☐ yes

☐ no

If YES: you are currently undergoing outpatient psychotherapeutic treatment

2.3.1 What type of treatment are you undergoing?

☐ *Individual psychotherapy*

☐ *Group psychotherapy*

☐ *ECT/ other stimulation methods*

☐ *Miscellaneous: ____*

2.3.2 How long have you been undergoing treatment?

For the interviewer: Indicate the treatment that was started first and continues.

Since _____________ (month/year)

2.4 Have you visited a doctor in the last 3 months because of your depression?

☐ yes

☐ no

If YES: you have visited a doctor

2.4.1 How often?

about ___________ times: with the following physicians

☐ *Family doctor*

☐ *Psychiatrist*

☐ *Other doctor: ______________________________________*

2.5 Have you been in outpatient psychotherapeutic individual or group treatment before?

For the interviewer: ask exactly! No quarterly contacts with the doctor!

☐ yes

☐ no

If YES: you have been in outpatient psychotherapeutic individual or group treatment before

2.5.1 How often have you been in outpatient psychotherapeutic individual or group treatment?

For the interviewer: If group and individual treatment were done at the same time and with the same therapist, this combination counts only as 1 time.

__________ times,

the last time from about _________ (month/year) to about ____________ (month/year)

2.6 Have you had an inpatient stay in a psychotherapeutic clinic in the past?

☐ yes

☐ no

If YES: you have had an inpatient stay in a psychotherapeutic clinic in the past

2.6.1 How often have you had an inpatient stay in a psychotherapeutic clinic?

__________ times,

the last time from about _________ (month/year) to about ____________ (month/year)

2.7 Have you been diagnosed with other mental disorders besides depression?

☐ yes

☐ no

If YES: you have been diagnosed with other mental disorders

2.7.1 Which?

1) ____

2) ____

3) ____

3. Questions about physical activity

3.1 Did you do any sports regularly before your illness?

☐ yes

☐ no

If YES: you did any sports before

3.1.1 Which sports? ______________________________

How many hours per week on average (across all sports)? ____________

3.2 Do you currently do sports - apart from the sports offered in our study?

☐ yes

☐ no

If YES: you currently do sports

3.2.1 Which sports? ______________________________

How many hours per week on average (across all sports)? ____________

3.3 What is your attitude towards physical activity?

☐ Physical activity is good for me.

☐ Physical activity is nothing for me.

4. Closure

4.1 We are now almost at the end of the telephone conversation. Is there anything else you would have liked to talk about or has been neglected in the conversation?

____

4.2 So that we can also contact you later: Is it foreseeable that your contact details will change (e.g. address, telephone)?

☐ yes

☐ no

If YES: new contact details

4.2.1 What are your new contact details? ______________________________

4.3 Arrange a date for the next telephone call in 10 weeks:

For the interviewer: Please check the CATI calendar for the next call

Thank you for the interview. We'll call you again in about 10 weeks. Would it be possible to make an appointment today? Or are there generally times during the day or days of the week when we can reach you easily?

____

4.4 Finally, I would like to ask you one last question. When you think about our next phone call in two months: what do you hope to be able to tell me then according to the therapy offer?

____

After hanging up, please assess the language competence of the interviewee.

4.5 Did you have the impression that the interview partner understood the questions (acoustically/lingually)?

☐ not at all (1)

☐ partially (2)

☐ completely (3)

4.6 Did you have the impression that the interviewee succeeded in answering the questions adequately?

☐ not at all (1)

☐ partially (2)

☐ completely (3)
